# Bis(2-amino-5-benzyl-3-eth­oxy­carbonyl-4,5,6,7-tetra­hydro­thieno[3,2-c]pyridin-5-ium) bis­(4-meth­oxy­phen­yl)di­phos­phon­ate

**DOI:** 10.1107/S1600536814003766

**Published:** 2014-02-22

**Authors:** Mehmet Akkurt, Joel T. Mague, Shaaban K. Mohamed, Sabry H. H. Younes, Mustafa R. Albayati

**Affiliations:** a Department of Physics, Faculty of Sciences, Erciyes University, 38039 Kayseri, Turkey; b Department of Chemistry, Tulane University, New Orleans, LA 70118, USA; c Chemistry and Environmental Division, Manchester Metropolitan University, Manchester M1 5GD, England; d Chemistry Department, Faculty of Science, Mini University, 61519 El-Minia, Egypt; e Department of Chemistry, Faculty of Science, Sohag University, 82524 Sohag, Egypt; f Kirkuk University, College of Science, Department of Chemistry, Kirkuk, Iraq

## Abstract

The asymmetric unit of the title salt, 2C_17_H_21_N_2_O_2_S^+^·C_14_H_14_O_7_P_2_^2−^, contains half of a centrosymmetric bis­(4-meth­oxy­phen­yl)di­phospho­nate anion and one 2-amino-5-benzyl-3-eth­oxy­carbonyl-4,5,6,7-tetra­hydro­thieno[3,2-*c*]pyri­din-5-ium cation. In the anion, the O atoms of the di­phospho­nate group are disordered over two positions with equal occupancies. In the cation, the ethyl group is disordered over two orientations with a refined occupancy ratio of 0.753 (5):0.247 (5), and the tetra­hydro­pyridinium ring adopts a distorted half-chair conformation. In the crystal, the ions are linked by C—H⋯O, N—H⋯O and C—H⋯S hydrogen bonds into a three-dimensional network.

## Related literature   

For medicinal applications of tetra­hydro­thieno­pyridines, see: Bernardino *et al.* (2006 ▶); Attaby *et al.* (1999 ▶); Kling *et al.* (2005 ▶); Baker & White (2009 ▶); Huber *et al.* (2009 ▶); Andersen *et al.* (2002 ▶); Boschellia *et al.* (2005 ▶); Tumeya *et al.* (2008 ▶). For a similar structure, see: Meng *et al.* (2011 ▶). For analysis of ring puckering, see: Cremer & Pople (1975 ▶).
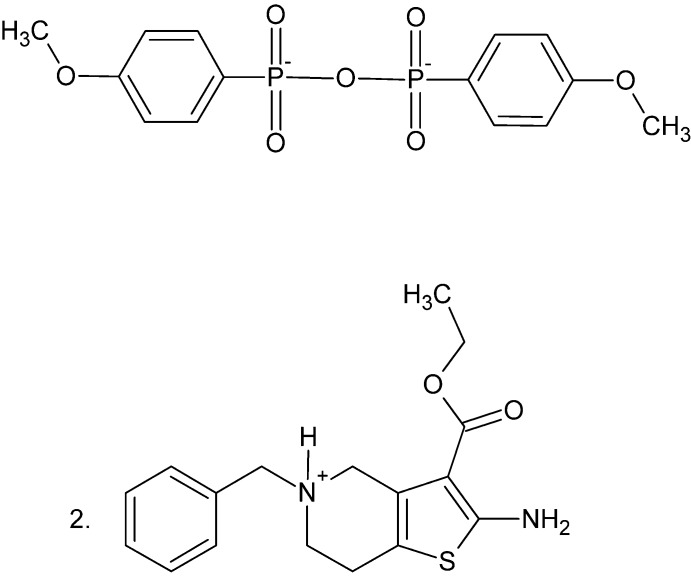


## Experimental   

### 

#### Crystal data  

2C_17_H_21_N_2_O_2_S^+^·C_14_H_14_O_7_P_2_^2−^
*M* *_r_* = 991.05
Monoclinic, 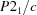

*a* = 14.9420 (9) Å
*b* = 10.8718 (7) Å
*c* = 16.0773 (10) Å
β = 114.3270 (8)°
*V* = 2379.8 (3) Å^3^
*Z* = 2
Mo *K*α radiation
μ = 0.24 mm^−1^
*T* = 150 K
0.23 × 0.19 × 0.05 mm

#### Data collection  

Bruker SMART APEX CCD diffractometer
Absorption correction: multi-scan (*SADABS*; Bruker, 2013 ▶) *T*_min_ = 0.81, *T*_max_ = 0.99
41576 measured reflections
5955 independent reflections
4481 reflections with *I* > 2σ(*I*)
*R*_int_ = 0.058

#### Refinement  

*R*[*F* ^2^ > 2σ(*F* ^2^)] = 0.049
*wR*(*F* ^2^) = 0.132
*S* = 1.04
5955 reflections
317 parameters
28 restraints
H atoms treated by a mixture of independent and constrained refinement
Δρ_max_ = 0.77 e Å^−3^
Δρ_min_ = −0.62 e Å^−3^

### 

Data collection: *APEX2* (Bruker, 2013 ▶); cell refinement: *SAINT* (Bruker, 2013 ▶); data reduction: *SAINT*; program(s) used to solve structure: *SHELXS97* (Sheldrick, 2008 ▶); program(s) used to refine structure: *SHELXL2013* (Sheldrick, 2008 ▶); molecular graphics: *ORTEP-3 for Windows* (Farrugia, 2012 ▶); software used to prepare material for publication: *PLATON* (Spek, 2009 ▶).

## Supplementary Material

Crystal structure: contains datablock(s) global, I. DOI: 10.1107/S1600536814003766/rz5105sup1.cif

Structure factors: contains datablock(s) I. DOI: 10.1107/S1600536814003766/rz5105Isup2.hkl

Supporting information file. DOI: 10.1107/S1600536814003766/rz5105Isup3.cml

CCDC reference: 987723

Additional supporting information:  crystallographic information; 3D view; checkCIF report

## Figures and Tables

**Table 1:** Hydrogen-bond geometry (Å, °)

*D*—H⋯*A*	*D*—H	H⋯*A*	*D*⋯*A*	*D*—H⋯*A*
N1—H1*N*⋯O4*A*	0.93 (2)	1.79 (3)	2.699 (4)	165 (2)
N1—H1*N*⋯O4*B*	0.93 (2)	1.72 (2)	2.641 (4)	171 (2)
N2—H2*N*⋯O5*A* ^i^	0.95 (3)	1.98 (3)	2.894 (4)	160 (3)
N2—H2*N*⋯O5*B* ^i^	0.95 (3)	1.77 (3)	2.686 (4)	162 (3)
N2—H3*N*⋯O1	0.84 (3)	2.11 (3)	2.761 (3)	135 (3)
C6—H6⋯O5*B* ^ii^	0.95	2.37	3.273 (4)	159
C7—H7*A*⋯O4*B* ^ii^	0.99	2.44	3.373 (4)	157
C7—H7*B*⋯S1^ii^	0.99	2.69	3.591 (2)	152
C8—H8*A*⋯O5*B* ^ii^	0.99	2.57	3.488 (4)	154
C18—H18*B*⋯O1^iii^	0.98	2.60	3.251 (4)	124
C20—H20⋯O1^iii^	0.95	2.59	3.524 (3)	168

Symmetry codes: (i) 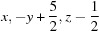; (ii) 
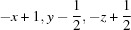
; (iii) 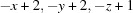.

**Fig. 1.**
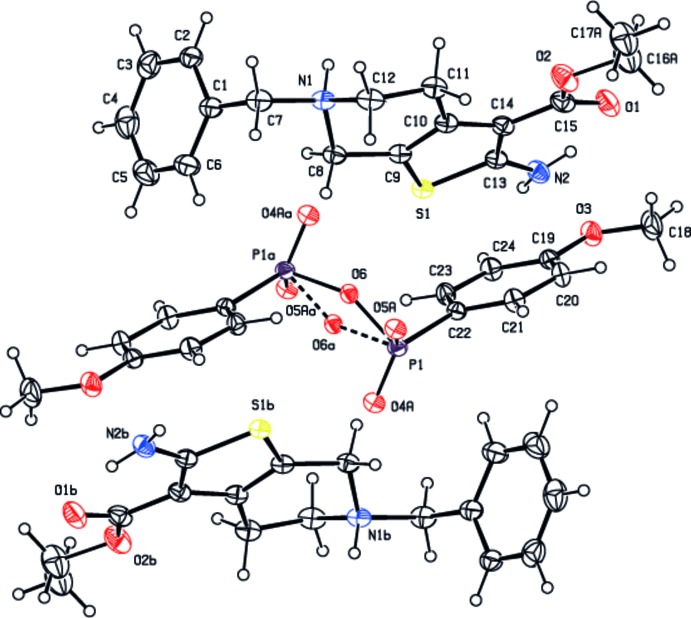
View of the title compound with displacement ellipsoids for non-H atoms drawn at the 30% probability level. Only one component of the disordered atoms (except atom O6) is shown. Symmetry codes: (a) 1-x, -y, 1-z; (b) x, -1+y, z.

## supplementary crystallographic information

### 1. Comment

Tetrahydrothieno pyridine-containing compounds are well known bioactive molecules due to their significant pharmaceutical and medicinal properties (Baker & White, 2009; Huber *et al.*, 2009; Andersen *et al.*, 2002; Boschellia *et al.*, 2005). They are used in the treatment of various stages of inflammation such as in chronic inflammatory rheumatism, degenerative rheumatism, oto-rhino-laryngology, stomatology, post-operative surgery and in traumatology (Kling *et al.*, 2005). They are also used in medicine as allosteric adenosine receptor modulators and in the treatment of adenosine-sensitive cardiac arrhythmias (Bernardino *et al.*, 2006; Attaby *et al.*, 1999; Tumeya *et al.*, 2008). As part of our on-going study to synthesize bio-hetero molecules, we report the synthesis and crystal structure of the title compound (I).

As seen in Fig. 1, the asymmetric unit of (I) consist of one cation and half of an anion. In the cation, the tetrahydropyridinium ring (N1/C8–C12) adopts a distorted half-chair conformation [puckering parameters (Cremer & Pople, 1975) are Q_T_ = 0.525 (2) Å, θ = 52.8 (2) ° and, φ = 341.7 (3) °]. The terminal phenyl ring (C1–C6) makes a dihedral angle of 90.02 (1)° with the thiophene ring (S1/C9/C10/C13/C14). All bond lengths and bond angles in (I) are within normal ranges when compared to those found in a similar structure (Meng *et al.*, 2011). In the crystal structure, the molecules are linked *via* intermolecular C—H···O, N—H···O and C—H···S hydrogen bonds (Table 1), forming three dimensional network.

### 2. Experimental

A mixture of 1 mmol (316 mg) ethyl 2-amino-5-benzyl-4,5,6,7-tetrahydrothieno[3,2-*c*]pyridine-3-carboxylate and 1 mmol (404.5 mg) of Lawesson's reagent (2,4-bis(4-methoxyphenyl)-1,3,2,4-dithiadiphosphetane 2,4-disulfide) in 30 ml acetonitrile was refluxed and monitored by TLC until completion (*ca* 6 h). The mixture was cooled to ambient temperature and the resulting solid product was collected by filtration, washed with diethyl ether and crystallized from ethanol in 84% yield. Plate-like yellow crystals suitable for X-ray analysis were prepared by slow evaporation of an ethanol solution of the title compound at room temperature over two days. M.p. 478 K.

### 3. Refinement

The N-bound H atoms were located in a difference Fourier map and refined isotropically with *U*_iso_(H) = 1.2*U*_eq_(N). All other H atoms were placed in geometrically idealized positions and refined using a riding model approximation, with C—H = 0.95–0.99 Å and with *U*_iso_(H) = 1.2*U*_eq_(C) or 1.5*U*_eq_(C) for methyl H atoms. In the anion, the O4 and O5 oxygen atoms are disordered over two sets of sites with equal site occupancies of 0.5, and the O6 atom is disordered about a centre of symmetry with site occupancy of 0.5. In the cation, the ethyl group is disordered over two orientations with refined occupancy ratio of 0.753 (5):0.247 (5). During the refinement the anisotropic displacement parameters of paired components of the disorder were restrained to be equivalent and approximately isotropic (EADP and ISOR commands in SHELX97-L).

### Crystal data

**Table:**

2C_17_H_21_N_2_O_2_S^+^·C_14_H_14_O_7_P_2_^2^^−^	*F*(000) = 1044
*M**_r_* = 991.05	*D*_x_ = 1.383 Mg m^−^^3^
Monoclinic, *P*2_1_/*c*	Mo *K*α radiation, λ = 0.71073 Å
Hall symbol: -P 2ybc	Cell parameters from 9946 reflections
*a* = 14.9420 (9) Å	θ = 2.3–28.3°
*b* = 10.8718 (7) Å	µ = 0.24 mm^−^^1^
*c* = 16.0773 (10) Å	*T* = 150 K
β = 114.3270 (8)°	Plate, pale yellow
*V* = 2379.8 (3) Å^3^	0.23 × 0.19 × 0.05 mm
*Z* = 2	

### Data collection

**Table:**

Bruker SMART APEX CCD diffractometer	5955 independent reflections
Radiation source: fine-focus sealed tube	4481 reflections with *I* > 2σ(*I*)
Graphite monochromator	*R*_int_ = 0.058
Detector resolution: 8.3660 pixels mm^-1^	θ_max_ = 28.4°, θ_min_ = 2.3°
φ and ω scans	*h* = −19→19
Absorption correction: multi-scan (*SADABS*; Bruker, 2013)	*k* = −14→14
*T*_min_ = 0.81, *T*_max_ = 0.99	*l* = −21→21
41576 measured reflections	

### Refinement

**Table:**

Refinement on *F*^2^	28 restraints
Least-squares matrix: full	Hydrogen site location: mixed
*R*[*F*^2^ > 2σ(*F*^2^)] = 0.049	H atoms treated by a mixture of independent and constrained refinement
*wR*(*F*^2^) = 0.132	*w* = 1/[σ^2^(*F*_o_^2^) + (0.0554*P*)^2^ + 1.6086*P*] where *P* = (*F*_o_^2^ + 2*F*_c_^2^)/3
*S* = 1.04	(Δ/σ)_max_ = 0.001
5955 reflections	Δρ_max_ = 0.77 e Å^−^^3^
317 parameters	Δρ_min_ = −0.62 e Å^−^^3^

### Special details

**Table:**

Geometry. Bond distances, angles *etc*. have been calculated using the rounded fractional coordinates. All su's are estimated from the variances of the (full) variance-covariance matrix. The cell e.s.d.'s are taken into account in the estimation of distances, angles and torsion angles
Refinement. Refinement on *F*^2^ for ALL reflections except those flagged by the user for potential systematic errors. Weighted *R*-factors *wR* and all goodnesses of fit *S* are based on *F*^2^, conventional *R*-factors *R* are based on *F*, with *F* set to zero for negative *F*^2^. The observed criterion of *F*^2^ > σ(*F*^2^) is used only for calculating -*R*-factor-obs *etc*. and is not relevant to the choice of reflections for refinement. *R*-factors based on *F*^2^ are statistically about twice as large as those based on *F*, and *R*-factors based on ALL data will be even larger.

### Fractional atomic coordinates and isotropic or equivalent isotropic displacement parameters (Å^2^)

**Table:**

	*x*	*y*	*z*	*U*_iso_*/*U*_eq_	Occ. (<1)
P1	0.57869 (4)	1.03372 (5)	0.46675 (4)	0.0305 (2)	
O3	0.86773 (11)	0.62642 (14)	0.61933 (12)	0.0427 (5)	
O4A	0.5173 (2)	1.0157 (3)	0.3725 (2)	0.0309 (5)	0.500
O4B	0.5512 (2)	1.0296 (3)	0.3642 (2)	0.0309 (5)	0.500
O5A	0.5998 (2)	1.1419 (3)	0.5154 (2)	0.0309 (5)	0.500
O5B	0.6358 (2)	1.1646 (3)	0.4900 (2)	0.0309 (5)	0.500
O6	0.52418 (18)	1.0374 (2)	0.53284 (18)	0.0274 (8)	0.500
C18	0.96967 (18)	0.6470 (3)	0.6554 (2)	0.0581 (10)	
C19	0.80643 (15)	0.72531 (19)	0.58665 (14)	0.0316 (6)	
C20	0.83752 (15)	0.8459 (2)	0.59149 (16)	0.0361 (6)	
C21	0.76781 (15)	0.93886 (19)	0.55603 (15)	0.0346 (6)	
C22	0.66850 (14)	0.91441 (18)	0.51714 (13)	0.0272 (5)	
C23	0.63917 (15)	0.7916 (2)	0.51452 (15)	0.0344 (6)	
C24	0.70699 (16)	0.69865 (19)	0.54824 (16)	0.0370 (7)	
S1	0.58537 (4)	1.08926 (4)	0.08978 (3)	0.0289 (1)	
O1	0.91131 (13)	1.09586 (18)	0.27696 (13)	0.0560 (7)	
O2	0.87989 (13)	0.92245 (18)	0.33537 (13)	0.0570 (6)	
N1	0.49957 (14)	0.85683 (15)	0.23756 (12)	0.0331 (5)	
N2	0.75816 (16)	1.19991 (18)	0.13051 (15)	0.0400 (6)	
C1	0.31772 (17)	0.8669 (2)	0.18867 (15)	0.0381 (7)	
C2	0.29320 (18)	0.9500 (2)	0.24134 (16)	0.0423 (7)	
C3	0.2121 (2)	1.0252 (3)	0.2017 (2)	0.0544 (10)	
C4	0.1555 (2)	1.0191 (3)	0.1092 (2)	0.0628 (11)	
C5	0.1785 (2)	0.9363 (3)	0.0564 (2)	0.0629 (10)	
C6	0.25792 (19)	0.8596 (3)	0.09563 (17)	0.0501 (8)	
C7	0.40835 (18)	0.7891 (2)	0.23008 (16)	0.0412 (7)	
C8	0.48723 (16)	0.91160 (19)	0.14823 (14)	0.0331 (6)	
C9	0.58213 (16)	0.96802 (17)	0.15942 (14)	0.0304 (6)	
C10	0.67162 (16)	0.94260 (18)	0.22578 (14)	0.0323 (6)	
C11	0.68191 (17)	0.8442 (2)	0.29481 (15)	0.0386 (7)	
C12	0.58719 (18)	0.77301 (19)	0.27059 (15)	0.0399 (7)	
C13	0.71049 (16)	1.11013 (18)	0.15399 (14)	0.0317 (6)	
C14	0.74755 (16)	1.02343 (19)	0.22349 (14)	0.0330 (6)	
C15	0.85221 (18)	1.0206 (2)	0.27950 (16)	0.0423 (7)	
C16A	0.9874 (3)	0.9227 (4)	0.3882 (4)	0.0673 (10)	0.753 (5)
C17A	1.0074 (3)	0.8018 (4)	0.4371 (3)	0.0673 (10)	0.753 (5)
C17B	1.0233 (8)	0.8259 (10)	0.3766 (6)	0.0673 (10)	0.247 (5)
C16B	0.9693 (9)	0.8858 (11)	0.4036 (11)	0.0673 (10)	0.247 (5)
H18A	1.00440	0.56880	0.67590	0.0870*	
H18B	0.98850	0.70380	0.70710	0.0870*	
H18C	0.98720	0.68280	0.60810	0.0870*	
H20	0.90560	0.86510	0.61870	0.0430*	
H21	0.78940	1.02150	0.55880	0.0420*	
H23	0.57110	0.77220	0.48900	0.0410*	
H24	0.68560	0.61580	0.54520	0.0440*	
H2	0.33250	0.95530	0.30510	0.0510*	
H2N	0.7183 (19)	1.261 (3)	0.0898 (18)	0.0480*	
H3N	0.816 (2)	1.207 (3)	0.1698 (18)	0.0480*	
H4	0.10050	1.07210	0.08190	0.0750*	
H3	0.19530	1.08130	0.23830	0.0650*	
H1N	0.5116 (18)	0.919 (2)	0.2803 (17)	0.0400*	
H7A	0.40010	0.71460	0.19220	0.0490*	
H7B	0.41660	0.76230	0.29170	0.0490*	
H8A	0.46820	0.84700	0.10060	0.0400*	
H8B	0.43500	0.97490	0.12920	0.0400*	
H11A	0.70120	0.88250	0.35560	0.0460*	
H11B	0.73470	0.78670	0.29840	0.0460*	
H12A	0.58000	0.71220	0.22240	0.0480*	
H12B	0.59020	0.72750	0.32500	0.0480*	
H16A	1.00820	0.99200	0.43200	0.0810*	0.753 (5)
H16B	1.02190	0.92850	0.34740	0.0810*	0.753 (5)
H17A	1.07800	0.79360	0.47450	0.1010*	0.753 (5)
H17B	0.98540	0.73480	0.39240	0.1010*	0.753 (5)
H17C	0.97190	0.79790	0.47650	0.1010*	0.753 (5)
H5	0.13940	0.93220	−0.00740	0.0750*	
H6	0.27220	0.80100	0.05900	0.0600*	
H16C	1.00540	0.96010	0.43580	0.0810*	0.247 (5)
H16D	0.95610	0.83530	0.44850	0.0810*	0.247 (5)
H17D	1.08390	0.80320	0.42890	0.1010*	0.247 (5)
H17E	1.03950	0.87620	0.33410	0.1010*	0.247 (5)
H17F	0.98900	0.75130	0.34540	0.1010*	0.247 (5)

### Atomic displacement parameters (Å^2^)

**Table:**

	*U*^11^	*U*^22^	*U*^33^	*U*^12^	*U*^13^	*U*^23^
P1	0.0343 (3)	0.0305 (3)	0.0281 (3)	0.0102 (2)	0.0143 (2)	0.0061 (2)
O3	0.0398 (9)	0.0337 (8)	0.0528 (10)	0.0139 (7)	0.0172 (8)	0.0100 (7)
O4A	0.0354 (11)	0.0229 (7)	0.0312 (8)	0.0003 (8)	0.0104 (7)	0.0002 (6)
O4B	0.0354 (11)	0.0229 (7)	0.0312 (8)	0.0003 (8)	0.0104 (7)	0.0002 (6)
O5A	0.0354 (11)	0.0229 (7)	0.0312 (8)	0.0003 (8)	0.0104 (7)	0.0002 (6)
O5B	0.0354 (11)	0.0229 (7)	0.0312 (8)	0.0003 (8)	0.0104 (7)	0.0002 (6)
O6	0.0248 (13)	0.0284 (14)	0.0272 (13)	0.0006 (10)	0.0090 (11)	−0.0027 (11)
C18	0.0409 (14)	0.0554 (16)	0.076 (2)	0.0206 (12)	0.0220 (14)	0.0182 (14)
C19	0.0343 (10)	0.0285 (10)	0.0324 (10)	0.0085 (8)	0.0143 (9)	0.0052 (8)
C20	0.0260 (10)	0.0333 (11)	0.0446 (12)	0.0019 (8)	0.0101 (9)	−0.0016 (9)
C21	0.0338 (11)	0.0246 (9)	0.0425 (12)	−0.0002 (8)	0.0128 (9)	−0.0027 (9)
C22	0.0297 (9)	0.0261 (9)	0.0246 (9)	0.0036 (8)	0.0101 (8)	−0.0001 (7)
C23	0.0273 (10)	0.0327 (11)	0.0403 (12)	−0.0019 (8)	0.0110 (9)	0.0037 (9)
C24	0.0375 (11)	0.0256 (10)	0.0459 (13)	0.0000 (9)	0.0153 (10)	0.0073 (9)
S1	0.0363 (3)	0.0210 (2)	0.0283 (2)	0.0004 (2)	0.0121 (2)	0.0003 (2)
O1	0.0402 (10)	0.0589 (12)	0.0560 (12)	−0.0101 (9)	0.0069 (9)	−0.0114 (9)
O2	0.0447 (10)	0.0558 (11)	0.0514 (11)	0.0127 (8)	0.0006 (8)	0.0041 (9)
N1	0.0489 (11)	0.0207 (8)	0.0293 (9)	−0.0042 (7)	0.0156 (8)	0.0009 (7)
N2	0.0404 (11)	0.0345 (10)	0.0403 (11)	−0.0099 (9)	0.0117 (9)	−0.0042 (9)
C1	0.0497 (13)	0.0345 (11)	0.0335 (11)	−0.0167 (10)	0.0205 (10)	−0.0019 (9)
C2	0.0475 (13)	0.0459 (13)	0.0367 (12)	−0.0138 (11)	0.0206 (11)	−0.0036 (10)
C3	0.0537 (16)	0.0502 (15)	0.0687 (19)	−0.0110 (12)	0.0347 (15)	−0.0037 (13)
C4	0.0482 (16)	0.0642 (19)	0.074 (2)	−0.0049 (14)	0.0231 (15)	0.0188 (16)
C5	0.0553 (17)	0.082 (2)	0.0424 (15)	−0.0182 (16)	0.0111 (13)	0.0112 (15)
C6	0.0586 (16)	0.0548 (15)	0.0355 (12)	−0.0186 (13)	0.0181 (12)	−0.0054 (11)
C7	0.0596 (15)	0.0274 (10)	0.0388 (12)	−0.0115 (10)	0.0224 (11)	0.0014 (9)
C8	0.0439 (12)	0.0271 (10)	0.0276 (10)	−0.0048 (9)	0.0139 (9)	0.0024 (8)
C9	0.0416 (11)	0.0210 (9)	0.0282 (10)	0.0026 (8)	0.0139 (9)	0.0004 (8)
C10	0.0409 (11)	0.0232 (9)	0.0317 (10)	0.0060 (8)	0.0138 (9)	−0.0019 (8)
C11	0.0492 (13)	0.0290 (10)	0.0334 (11)	0.0087 (9)	0.0127 (10)	0.0053 (9)
C12	0.0596 (14)	0.0215 (9)	0.0351 (11)	0.0051 (9)	0.0161 (11)	0.0041 (9)
C13	0.0388 (11)	0.0239 (9)	0.0327 (11)	−0.0001 (8)	0.0149 (9)	−0.0076 (8)
C14	0.0378 (11)	0.0265 (10)	0.0318 (10)	0.0035 (8)	0.0115 (9)	−0.0059 (8)
C15	0.0428 (13)	0.0408 (13)	0.0361 (12)	0.0074 (10)	0.0090 (10)	−0.0073 (10)
C16A	0.0518 (16)	0.0641 (19)	0.0685 (19)	0.0069 (13)	0.0073 (13)	0.0263 (15)
C17A	0.0518 (16)	0.0641 (19)	0.0685 (19)	0.0069 (13)	0.0073 (13)	0.0263 (15)
C17B	0.0518 (16)	0.0641 (19)	0.0685 (19)	0.0069 (13)	0.0073 (13)	0.0263 (15)
C16B	0.0518 (16)	0.0641 (19)	0.0685 (19)	0.0069 (13)	0.0073 (13)	0.0263 (15)

### Geometric parameters (Å, º)

**Table:**

S1—C9	1.743 (2)	C1—C6	1.393 (3)
S1—C13	1.739 (2)	C2—C3	1.381 (4)
P1—O4A	1.425 (3)	C3—C4	1.377 (4)
P1—O5B	1.622 (3)	C4—C5	1.375 (4)
P1—O6	1.583 (3)	C5—C6	1.373 (4)
P1—C22	1.801 (2)	C8—C9	1.486 (3)
P1—O6^i^	1.723 (3)	C9—C10	1.352 (3)
P1—O4B	1.527 (3)	C10—C11	1.503 (3)
P1—O5A	1.375 (3)	C10—C14	1.448 (3)
O3—C18	1.407 (4)	C11—C12	1.517 (4)
O3—C19	1.369 (3)	C13—C14	1.391 (3)
O5A—O6	1.705 (4)	C14—C15	1.448 (4)
O1—C15	1.217 (3)	C16A—C17A	1.497 (6)
O2—C16A	1.476 (6)	C16B—C17B	1.246 (19)
O2—C15	1.346 (3)	C2—H2	0.9500
O2—C16B	1.393 (16)	C3—H3	0.9500
N1—C8	1.494 (3)	C4—H4	0.9500
N1—C7	1.510 (3)	C5—H5	0.9500
N1—C12	1.501 (3)	C6—H6	0.9500
N2—C13	1.351 (3)	C7—H7A	0.9900
N1—H1N	0.93 (2)	C7—H7B	0.9900
N2—H2N	0.95 (3)	C8—H8A	0.9900
N2—H3N	0.84 (3)	C8—H8B	0.9900
C19—C20	1.382 (3)	C11—H11A	0.9900
C19—C24	1.385 (3)	C11—H11B	0.9900
C20—C21	1.394 (3)	C12—H12A	0.9900
C21—C22	1.378 (3)	C12—H12B	0.9900
C22—C23	1.401 (3)	C16A—H16A	0.9900
C23—C24	1.375 (3)	C16A—H16B	0.9900
C18—H18A	0.9800	C16B—H16C	0.9900
C18—H18B	0.9800	C16B—H16D	0.9900
C18—H18C	0.9800	C17A—H17C	0.9800
C20—H20	0.9500	C17A—H17A	0.9800
C21—H21	0.9500	C17A—H17B	0.9800
C23—H23	0.9500	C17B—H17D	0.9800
C24—H24	0.9500	C17B—H17E	0.9800
C1—C2	1.387 (3)	C17B—H17F	0.9800
C1—C7	1.500 (4)		
			
C9—S1—C13	91.43 (11)	S1—C9—C8	120.79 (16)
O4A—P1—O5B	114.38 (17)	S1—C9—C10	112.42 (18)
O4A—P1—O6	115.68 (17)	C9—C10—C11	119.7 (2)
O4A—P1—O5A	128.22 (19)	C11—C10—C14	127.5 (2)
O4A—P1—O6^i^	75.99 (16)	C9—C10—C14	112.75 (19)
O4B—P1—O5A	122.41 (18)	C10—C11—C12	111.89 (19)
O4B—P1—O5B	99.01 (16)	N1—C12—C11	111.51 (17)
O4B—P1—O6	137.86 (16)	N2—C13—C14	129.3 (2)
O4B—P1—C22	106.48 (14)	S1—C13—C14	111.32 (17)
O4B—P1—O6^i^	98.48 (15)	S1—C13—N2	119.36 (17)
O5A—P1—O6	69.98 (16)	C10—C14—C13	112.0 (2)
O5A—P1—C22	113.97 (15)	C10—C14—C15	128.92 (19)
O5A—P1—O6^i^	112.18 (16)	C13—C14—C15	119.0 (2)
O5B—P1—O6	100.98 (14)	O1—C15—O2	121.9 (2)
O5B—P1—C22	107.72 (13)	O1—C15—C14	125.3 (2)
O5B—P1—O6^i^	141.12 (14)	O2—C15—C14	112.8 (2)
O6—P1—C22	102.14 (11)	O2—C16A—C17A	103.6 (4)
O4A—P1—C22	114.41 (15)	O2—C16B—C17B	115.1 (12)
O6^i^—P1—C22	100.12 (11)	C1—C2—H2	120.00
O6—P1—O6^i^	45.77 (13)	C3—C2—H2	120.00
C18—O3—C19	118.2 (2)	C2—C3—H3	120.00
P1—O5A—O6	60.75 (15)	C4—C3—H3	120.00
P1—O6—O5A	49.27 (13)	C3—C4—H4	120.00
P1—O6—P1^i^	134.23 (16)	C5—C4—H4	120.00
P1^i^—O6—O5A	162.2 (2)	C4—C5—H5	120.00
C15—O2—C16A	110.2 (3)	C6—C5—H5	120.00
C15—O2—C16B	133.2 (6)	C1—C6—H6	120.00
C8—N1—C12	109.04 (19)	C5—C6—H6	120.00
C7—N1—C8	111.58 (18)	N1—C7—H7A	109.00
C7—N1—C12	111.06 (17)	N1—C7—H7B	109.00
C8—N1—H1N	109.4 (15)	C1—C7—H7A	109.00
C12—N1—H1N	107.8 (17)	C1—C7—H7B	109.00
C7—N1—H1N	107.9 (18)	H7A—C7—H7B	108.00
C13—N2—H2N	116.4 (19)	N1—C8—H8A	110.00
C13—N2—H3N	111 (2)	N1—C8—H8B	110.00
H2N—N2—H3N	128 (3)	C9—C8—H8A	110.00
O3—C19—C20	124.5 (2)	C9—C8—H8B	110.00
O3—C19—C24	115.60 (19)	H8A—C8—H8B	108.00
C20—C19—C24	119.8 (2)	C10—C11—H11A	109.00
C19—C20—C21	119.2 (2)	C10—C11—H11B	109.00
C20—C21—C22	122.0 (2)	C12—C11—H11A	109.00
P1—C22—C23	120.47 (17)	C12—C11—H11B	109.00
P1—C22—C21	121.92 (16)	H11A—C11—H11B	108.00
C21—C22—C23	117.56 (19)	N1—C12—H12A	109.00
C22—C23—C24	121.2 (2)	N1—C12—H12B	109.00
C19—C24—C23	120.2 (2)	C11—C12—H12A	109.00
O3—C18—H18B	110.00	C11—C12—H12B	109.00
O3—C18—H18C	109.00	H12A—C12—H12B	108.00
H18A—C18—H18B	110.00	O2—C16A—H16A	111.00
O3—C18—H18A	110.00	O2—C16A—H16B	111.00
H18A—C18—H18C	109.00	C17A—C16A—H16A	111.00
H18B—C18—H18C	109.00	C17A—C16A—H16B	111.00
C21—C20—H20	120.00	H16A—C16A—H16B	109.00
C19—C20—H20	120.00	O2—C16B—H16D	109.00
C20—C21—H21	119.00	C17B—C16B—H16C	108.00
C22—C21—H21	119.00	C17B—C16B—H16D	108.00
C24—C23—H23	119.00	H16C—C16B—H16D	107.00
C22—C23—H23	119.00	O2—C16B—H16C	109.00
C23—C24—H24	120.00	H17A—C17A—H17C	109.00
C19—C24—H24	120.00	H17B—C17A—H17C	110.00
C2—C1—C6	118.6 (2)	C16A—C17A—H17A	109.00
C6—C1—C7	120.5 (2)	C16A—C17A—H17B	109.00
C2—C1—C7	120.9 (2)	C16A—C17A—H17C	109.00
C1—C2—C3	120.4 (2)	H17A—C17A—H17B	109.00
C2—C3—C4	120.1 (3)	C16B—C17B—H17D	110.00
C3—C4—C5	120.0 (3)	C16B—C17B—H17E	109.00
C4—C5—C6	120.2 (3)	C16B—C17B—H17F	109.00
C1—C6—C5	120.6 (3)	H17D—C17B—H17E	109.00
N1—C7—C1	112.38 (18)	H17D—C17B—H17F	110.00
N1—C8—C9	108.32 (18)	H17E—C17B—H17F	109.00
C8—C9—C10	126.54 (19)		
			
C13—S1—C9—C10	−1.85 (18)	C7—N1—C8—C9	−175.70 (17)
C13—S1—C9—C8	172.78 (18)	C8—N1—C12—C11	67.0 (2)
C9—S1—C13—C14	2.30 (17)	C12—N1—C7—C1	−174.44 (18)
C9—S1—C13—N2	−179.77 (19)	C8—N1—C7—C1	−52.6 (2)
C22—P1—O5A—O6	−94.76 (15)	O3—C19—C24—C23	179.1 (2)
O5B—P1—O5A—O6	−179.4 (4)	C24—C19—C20—C21	−1.1 (3)
O4B—P1—O6—O5A	−116.6 (2)	C20—C19—C24—C23	0.3 (3)
O6^i^—P1—O5A—O6	18.13 (17)	O3—C19—C20—C21	−179.7 (2)
O4A—P1—O6—O5A	−123.7 (2)	C19—C20—C21—C22	0.8 (3)
O6^i^—P1—O6—O5A	−156.3 (2)	C20—C21—C22—P1	−177.16 (18)
O4A—P1—O6—P1^i^	32.6 (3)	C20—C21—C22—C23	0.2 (3)
O4B—P1—O6—P1^i^	39.7 (3)	C21—C22—C23—C24	−1.0 (3)
O5A—P1—O6—P1^i^	156.3 (3)	P1—C22—C23—C24	176.45 (18)
O5B—P1—O6—P1^i^	156.6 (2)	C22—C23—C24—C19	0.7 (3)
O5B—P1—O6—O5A	0.31 (19)	C7—C1—C6—C5	−175.7 (3)
C22—P1—O6—O5A	111.34 (16)	C2—C1—C7—N1	−82.4 (3)
O4B—P1—O5A—O6	134.7 (2)	C7—C1—C2—C3	177.0 (3)
O4B^i^—P1^i^—O6—P1	154.3 (2)	C6—C1—C7—N1	95.7 (3)
O5A^i^—P1^i^—O6—P1	24.1 (3)	C2—C1—C6—C5	2.4 (4)
O5B^i^—P1^i^—O6—P1	38.4 (3)	C6—C1—C2—C3	−1.1 (4)
O6^i^—P1^i^—O6—P1	0.02 (18)	C1—C2—C3—C4	−0.7 (4)
C22^i^—P1^i^—O6—P1	−97.2 (2)	C2—C3—C4—C5	1.3 (5)
O4A—P1—C22—C23	−56.9 (2)	C3—C4—C5—C6	−0.1 (5)
O4B—P1—C22—C23	−79.8 (2)	C4—C5—C6—C1	−1.8 (5)
O5A—P1—C22—C23	142.2 (2)	N1—C8—C9—S1	−153.96 (15)
O5B—P1—C22—C23	174.75 (19)	N1—C8—C9—C10	19.9 (3)
O6—P1—C22—C23	68.9 (2)	S1—C9—C10—C11	177.33 (16)
O6^i^—P1—C22—C23	22.2 (2)	S1—C9—C10—C14	0.9 (2)
O6—P1—C22—C21	−113.8 (2)	C8—C9—C10—C11	3.1 (3)
O6^i^—P1—C22—C21	−160.46 (19)	C8—C9—C10—C14	−173.3 (2)
O5A—P1—C22—C21	−40.5 (2)	C9—C10—C11—C12	8.0 (3)
O4A^i^—P1^i^—O6—P1	150.0 (3)	C14—C10—C11—C12	−176.1 (2)
O4B—P1—C22—C21	97.5 (2)	C9—C10—C14—C13	0.8 (3)
C22—P1—O6—P1^i^	−92.4 (2)	C9—C10—C14—C15	−176.2 (2)
O5B—P1—C22—C21	−7.9 (2)	C11—C10—C14—C13	−175.2 (2)
O4A—P1—O5A—O6	107.5 (2)	C11—C10—C14—C15	7.8 (4)
O4A—P1—C22—C21	120.4 (2)	C10—C11—C12—N1	−42.3 (3)
O6^i^—P1—O6—P1^i^	0.00 (16)	S1—C13—C14—C10	−2.2 (2)
C18—O3—C19—C24	177.2 (2)	N2—C13—C14—C15	−2.5 (4)
C18—O3—C19—C20	−4.1 (3)	S1—C13—C14—C15	175.15 (17)
C16A—O2—C15—O1	−0.6 (4)	N2—C13—C14—C10	−179.9 (2)
C16A—O2—C15—C14	178.1 (3)	C10—C14—C15—O1	−175.9 (2)
C15—O2—C16A—C17A	−173.1 (3)	C10—C14—C15—O2	5.5 (3)
C7—N1—C12—C11	−169.64 (18)	C13—C14—C15—O1	7.3 (4)
C12—N1—C8—C9	−52.7 (2)	C13—C14—C15—O2	−171.4 (2)

Symmetry code: (i) −*x*+1, −*y*+2, −*z*+1.

### Hydrogen-bond geometry (Å, º)

**Table:**

*D*—H···*A*	*D*—H	H···*A*	*D*···*A*	*D*—H···*A*
N1—H1*N*···O4*A*	0.93 (2)	1.79 (3)	2.699 (4)	165 (2)
N1—H1*N*···O4*B*	0.93 (2)	1.72 (2)	2.641 (4)	171 (2)
N2—H2*N*···O5*A*^ii^	0.95 (3)	1.98 (3)	2.894 (4)	160 (3)
N2—H2*N*···O5*B*^ii^	0.95 (3)	1.77 (3)	2.686 (4)	162 (3)
N2—H3*N*···O1	0.84 (3)	2.11 (3)	2.761 (3)	135 (3)
C6—H6···O5*B*^iii^	0.95	2.37	3.273 (4)	159
C7—H7*A*···O4*B*^iii^	0.99	2.44	3.373 (4)	157
C7—H7*B*···S1^iii^	0.99	2.69	3.591 (2)	152
C8—H8*A*···O5*B*^iii^	0.99	2.57	3.488 (4)	154
C18—H18*B*···O1^iv^	0.98	2.60	3.251 (4)	124
C20—H20···O1^iv^	0.95	2.59	3.524 (3)	168

Symmetry codes: (ii) *x*, −*y*+5/2, *z*−1/2; (iii) −*x*+1, *y*−1/2, −*z*+1/2; (iv) −*x*+2, −*y*+2, −*z*+1.
